# Draft Genome of Janthinobacterium sp. RA13 Isolated from Lake Washington Sediment

**DOI:** 10.1128/genomeA.01588-14

**Published:** 2015-02-12

**Authors:** Tami L. McTaggart, Nicole Shapiro, Tanja Woyke, Ludmila Chistoserdova

**Affiliations:** a Department of Chemical Engineering, University of Washington, Seattle, Washington, USA; b DOE Joint Genome Institute, Walnut Creek, California, USA

## Abstract

Sequencing the genome of Janthinobacterium sp. RA13 from Lake Washington sediment is announced. From the genome content, a versatile life-style is predicted, but not *bona fide* methylotrophy. With the availability of its genomic sequence, Janthinobacterium sp. RA13 presents a prospective model for studying microbial communities in lake sediments.

## GENOME ANNOUNCEMENT

When natural microbial communities from Lake Washington sediment are incubated under the atmosphere of methane, simple, semi-stable communities are formed, consisting of *bona fide* methanotroph species and of nonmethanotrophic satellite species. One of the types found to persist in such methane-fed microcosms is the Janthinobacterium species (1). Strain Janthinobacterium sp. RA13 was isolated from such an enrichment culture that was incubated at 10°C in a minimal salts medium, with multiple transfers and dilutions, for approximately 18 months (1), by plating onto Nutrient Broth (NB) agar medium (Difco). Axenic culture was obtained by selecting a single colony, followed by multiple re-streaking onto fresh NB plates. The culture features a typical violet color caused by violacein (2).

The draft genome of Janthinobacterium sp. RA13 was generated at the DOE Joint genome Institute (JGI), Walnut Creek, CA, USA using Pacific Biosciences (PacBio) sequencing technology (3). All general aspects of library construction and sequencing performed at the JGI can be found at http://www.jgi.doe.gov. The raw reads were assembled using HGAP (version 2.2.0.p1) (4). The final draft assembly contains one contig, totaling 6,421,258 bp in size. Genes were identified using Prodigal (5), followed by a round of manual curation using GenePRIMP (6). The predicted coding sequences (CDSs) were translated and used to search the National Center for Biotechnology Information (NCBI) nonredundant database, UniProt, TIGRFam, Pfam, KEGG, COG, and InterPro databases. The tRNAScanSE tool (7) was used to find tRNA genes, whereas rRNA genes were found by searches against models of the rRNA genes built from SILVA (8). Other noncoding RNAs such as the RNA components of the protein secretion complex and the RNase P were identified by searching the genome for the corresponding Rfam profiles using Infernal (http://infernal.janelia.org). Additional gene prediction analysis and manual functional annotation was performed within the Integrated Microbial Genomes (IMG) platform (http://img.jgi.doe.gov) developed by the JGI (9).

From the genome content, a versatile lifestyle can be predicted for Janthinobacterium sp. RA13, including the potential for anaerobic metabolism linked to denitrification. However, with the exception of the C1 transfer pathway linked to tetrahydrofolate, no traditional methylotrophy pathways (10) are identifiable. With the availability of its genomic sequence, Janthinobacterium sp. RA13 presents a prospective model for studying microbial communities in lake sediments.

### Nucleotide sequence accession number. 

The genome sequence has been deposited in GenBank under the accession no. JQNP01000001.
